# Improved Hydrophobic, UV Barrier and Antibacterial Properties of Multifunctional PVA Nanocomposite Films Reinforced with Modified Lignin Contained Cellulose Nanofibers

**DOI:** 10.3390/polym14091705

**Published:** 2022-04-22

**Authors:** Yujie Li, Yifan Chen, Qiang Wu, Jingda Huang, Yadong Zhao, Qian Li, Siqun Wang

**Affiliations:** 1 College of Chemistry and Materials Engineering, Zhejiang A&F University, Hangzhou 311300, China; lyjzafu@163.com (Y.L.); yfchen.28425@foxmail.com (Y.C.); wuqiang@zafu.edu.cn (Q.W.); hjd1015@163.com (J.H.); 2 School of Food and Pharmacy, Zhejiang Ocean University, Zhoushan 316022, China; yadong@kth.se; 3 Center for Renewable Carbon, University of Tennessee, Knoxville, TN 37996, USA

**Keywords:** LCNF, PVA, hydrophobicity, antibacterial property, UV shielding

## Abstract

In this study, we reported PVA nanocomposite films enhanced by polyethyleneimine (PEI)-lignin contained cellulose nanofibers (LCNFs) via the solvent casting method. An easy and available method was preformed to prepare LCNFs using a supermasscolloider from unbleached bamboo waste after a mild alkaline pretreatment. The results demonstrate that LCNF–PEI can greatly improve mechanical, hydrophobic, anti-UV shielding and antibacterial properties of the composite films. The tensile strength of LPP1 film was improved to 54.56 MPa, which was higher than 39.37 MPa of PVA film. The water contact angle of films increased from 35° to 104° with an increase in LCNF content from 0 to 6 wt%. Meanwhile, the nanocomposite film demonstrated the effect of full shielding against ultraviolet light when the amount of LCNF–PEI reached 6 wt%. The addition of LCNF–PEI endowed excellent antibacterial activity (against *S. aureus* and *E. coli*), which indicated potential applications in the packaging field.

## 1. Introduction

With the decrease in ozone content in the stratosphere, ultraviolet (UV) radiation from the solar spectrum has a ubiquitous harmful impact on human health and other biological systems. Developing UV-shielding packaging materials have been gradually gaining attention [1,2]. Owing to the biocompatible, natural polymer-based biomaterials, such as chitosan, collagen and zein, they may become a great achievement in the field of green packaging [3,4,5]. Polyvinyl alcohol (PVA) is a kind of linear polymer with bio-compatibility, biodegradability, non-toxicity and good mechanical properties [6,7]. Because of its excellent film-forming and barrier ability, PVA is widely used for medical treatment, emulsifying, water purification and food packaging [8,9]. The usage of PVA materials for packaging can reduce ecological waste and eliminate the problems of environmental pollution. However, PVA itself has a poor resistance to UV light, which limits its application as UV shielding packaging materials. In order to part from photo-oxidation effects of excessive ultraviolet radiation, adding UV shielding agents to PVA-based films is an effective way. The traditional metal oxidant (such as TiO_2_ and ZnO) have excellent reflection and scattering characteristics for UV light, but their photocatalytic activity easily induces the degradation of the polymer matrix and increases the risk of cancer [10,11]. Moreover, the service life of PVA films are easily affected by its hydrophilicity, which leads to low mechanical properties and a rapid growth of microorganisms in high humidity [12]. Therefore, developing hydrophobic, anti-UV shielding and antibacterial PVA packaging material still remains a challenge.

Lignin, the second largest biomass resource in plants, is an amorphous biopolymer with a highly branched complex structure [13,14,15]. Due to its many aromatic rings and complex three-dimensional network structure, it can effectively protect the oxidative free radical damage caused by UV irradiation and has the performance of UV absorption. Moreover, the highly branched and cross-linked lignin molecules induced a complex three-dimensional network, which can serve as an excellent hydrophobic agent in the polymeric matrix [16,17,18,19,20]. Lignocellulose nanofiber (LCNF), containing lignin and insoluble hemicellulose along with cellulose fibers, is a nano-scale high value-added product of biomass [21,22,23,24]. Similar with cellulose nanofiber (CNF), LCNF is also used as a reinforcement filler for the preparation of nanocomposites because of its good mechanical property, high stiffness and relative surface area [25,26]. Traditionally, removal of lignin and hemicellulose from lignocellulose in the preparation of CNF increases the product cost and causes environmental issues [27,28]. Recent studies indicated that the presence of residual lignin in nanocellulose demonstrated potential advantages such as bacteriostasis, high thermal stability and the capacity of water blocking, and so on [29]. Bian et al. prepared a natural lignocellulosic nanofibril (LCNF) film with a lignin content of 4.89–15.68% by vacuum filtration and pressing process, which exhibited excellent thermal stability and UVA and UVB blocking [30]. Wang et al. dispersed lignin-containing cellulose nanofibers (LCNFs) into polylactic acid (PLA) and found that the presence of lignin can improve the intermolecular interaction and compatibility [31]. To explore its potential application, the chemical modification of LCNF provides an efficient approach to improve the utilization of LCNF in polymer materials.

Polyethyleneimine (PEI) is a cationic polyelectrolyte with high charge density and a large number of amine groups [32,33]. PEI had excellent bacteriostatic effect on gram-negative escherichia coli and gram-positive staphylococcus. Many researchers pay great attention to amine-based polymers such as polyethyleneimine, haloamine and chitosan as bactericides for bacteriostasis and for the elimination of harmful microorganisms. The modification of LCNF with PEI could prolong shelf life and enhance storage quality in food and non-food industries, which have potential applications for UV shielding packaging.

The utilization rate of bamboo in the traditional production and processing process is less than 40%, and a large amount of bamboo waste cannot be utilized effectively. In order to improve the comprehensive utilization rate of bamboo resources and enrich the function of polyvinyl alcohol film, we reported a facile method to fabricate a series of biodegradable LCNF-PVA nanocomposite films by the solvent cast method with high strength, excellent anti-UV shielding and antibacterial properties. First, LCNFs were obtained from bamboo waste under mild alkaline pretreatment combined with an ultrafine friction grinder (supermasscolloider). Then, the obtained LCNF was effectively modified with PEI to fabricate LCNF–PEI. Finally, the modified LCNF was introduced into the PVA matrix to prepare nanocomposite packaging films. The morphology, mechanical performance, thermal stability, anti-UV shielding, antimicrobial and surface hydrophobicity of the resulting films were explored. This process provides an efficient approach to improve the utilization of LCNF in the fields of photosensitive material coating, pharmaceutical and food packaging materials.

## 2. Materials and Methods

### 2.1. Materials

Bamboo waste was collected from the Anji, Zhejiang Province, China and then smashed under 40 mesh. Sodium hydroxide, polyethyleneimine (PEI, *M_w_* 70,000, 50% in water), epichlorohydrin (99.5%), polyvinyl alcohol (1750 ± 50) and lauryl sodium sulfate were purchased from Macklin Biochemical Co., Ltd., Shanghai, China and implemented without further purification.

### 2.2. Preparation of LCNF and LCNF–PEI Suspension

Bamboo sample is dried and stored after neutral detergent (3 wt% lauryl sodium sulfate) extraction for further experiments. The pretreated bamboo sample (10 g) was immersed into 200 mL of NaOH aqueous solution (1 wt%) at 25 °C for 6 h under continuous shaking. After the treatment, the solid residues were washed to neutrality with deionized water and then nanofibrillated using an ultra-fine friction grinder (supermasscolloider, model MKCA6-5JR, Masuko Sangyo Co., Ltd., Saitama, Japan) at a rotor speed of 1500 rpm. The grinding treatment was performed with a clearance gauge of −15 between the two grind stones corresponding to 150 μm from the zero position. The LCNF (62.6% cellulose, 19.7% hemicellulose, 16.7% lignin) suspension with solid content 0.84 wt% was obtained after 10 cycles.

**Figure 1:**
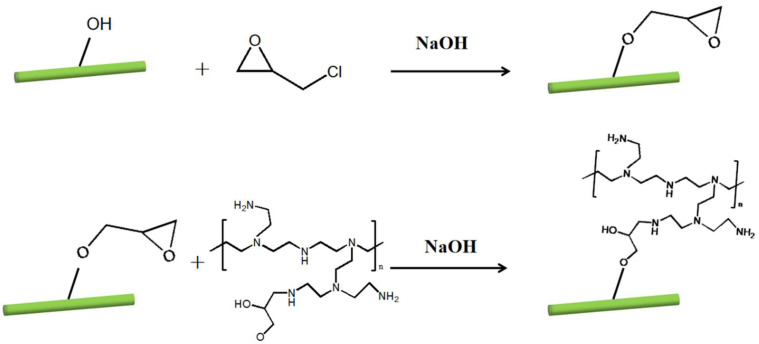
Schematic preparation of the LCNF–PEI.

The mechanism of the successful graft of PEI on the LCNF is shown in Figure 1. A certain amount of LCNF was stirred in the NaOH aqueous solution. A total of 1.25 mL of epichlorohydrin (EPI) as a crosslinker was slowly dropped into a certain amount of LCNF suspension at 40 °C for 3 h. PEI was then added dropwise and reacted for 2 h. The mixture was neutralized with acetic acid and dialyzed with regenerated cellulose tubes (*M_w_* cutoff 8000, Spectrum, Elisno, USA) against distilled water for 7 days. The resulting suspension was labeled as LCNF–PEI.

### 2.3. Preparation of LCNF–PEI/PVA Nanocomposite Film

**Figure 2:**
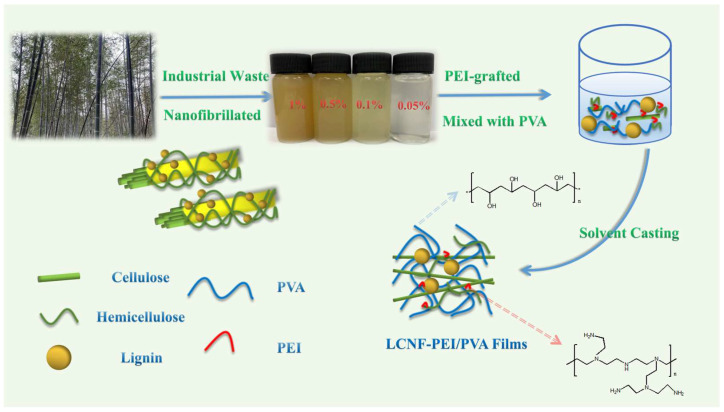
Schematic preparation of LPP nanocomposite films.

LCNF–PEI/PVA nanocomposite films (LPPs) were prepared by the solvent casting method [12] as the following: 10 g PVA sample was dissolved in 90 g of deionized water at a 95 °C oil bath under stirring for 2.5 h. Next, an appropriate amount of LCNF–PEI suspension was dispersed into the 10 wt% PVA solution according to the different ratio and fully stirred at 95 °C for 30 min until homogenized. Then, ultrasonic treatment was used for the further dispersion of LCNF in the matrix. Finally, the resulting mixture was degassed, poured into a glass plate and dried at 45 °C in an oven. The proportion of LCNF–PEI in PVA films was set at 1 wt%, 2 wt%, 4 wt% and 6 wt%, and the corresponding films were labeled as LPP1, LPP2, LPP4 and LPP6, respectively. As controlled samples, pure PVA film and the film CPP1 (the proportion of CNF without lignin in the PVA film was 1 wt%) were also prepared through the same fabrication process. The fabrication process for LCNF–PEI/PVA nanocomposite films is schematically summarized in Figure 2.

### 2.4. Characterization

Transmission electron microscopy (TEM) images were performed on JEM-2300 TEM (JEOL Ltd., Tokyo, Japan) with an accelerating voltage of 120 kV. The suspension of LCNF was dropped onto copper grids and allowed to dry at room temperature. 

Scanning electron microscopy (SEM) measurements were carried out on a FESEM (Merlin of Zeiss, Germany) by using an accelerating voltage of 15 KV. The surface and cross sections of the films were coated with gold for SEM observation. 

The modification of LCNF was characterized at the scanning range from 4000 to 400 cm^−1^ by using FTIR (Nicolet 6700, Thermo Fisher Scientific, Waltham, MA, USA). 

Water uptakes (*WU*_∞_) of the films in distilled water were determined as the following. The specimens with a typical size of 5 cm × 5 cm (length × width) were dried in a vacuum at 105 °C. After weighing, the films were immersed into distilled water for 48 h at room temperature until the equilibrium state was reached and wiped off the surface water droplets, followed immediately by weighing. Each experiment was repeated three times and the average values were calculated according to Equation (1).

*WU*_∞_ = (*W*_∞_ − *W*_0_)/*W*_0_ × 100% (1)

where *W*_∞_ and *W*_0_ are the equilibrium weight and initial weight of the samples, respectively.

To check the shielding effect of the films against UV radiation, the transmittance of the films was measured by ultraviolet spectrophotometry (UV-2400, Sunny hengping instrument, Shanghai, China) at a wavelength range from 200 to 800 nm. 

The water contact angle (WCA) of the samples were tested using a contact angle analyzer (TBU 100, Zhongchen digital technic apparatus co., Ltd., Shanghai, China). Images of the droplets on the films were recorded at different times after the drop touched the film surface.

Thermal stability was explored by TGA (TG209F1, Netzsch, Bavaria, Germany) with a range from 35 to 800 °C at a heating rate of 20 °C/min under the *N*_2_ atmosphere. All samples were cut into particle-like size and then vacuum-dried for 24 h at room temperature before the measurement. 

A differential scanning calorimeter (DSC, TA Instruments Q2000, New Castle, DE, USA) was used to perform thermal stability under a nitrogen atmosphere from 40 °C to 240 °C with a scanning rate of 10 °C/min. Before the test, the sample was heated to 240 °C and then cooled to 30 °C at a cooling rate of 20 °C/min to eliminate thermal history.

The tensile strength (*σ_b_*), elongation at break (*ε_b_*) and Young’s modulus of the films were determined with a universal testing machine (CMT 6503, Shenzhen SANS Test Machine Co., Ltd., Shenzhen, China) with a tensile rate of 5 mm/min at room temperature according to ISO 6293-1986 (E). Tensile tests were carried out at room temperature. The samples were stored in the dryer and taken out before the tests in the vacuum oven for the dry treatment.

In order to analyze the antibacterial performance of the films, this study adopted the agar diffusion method [34]. The inhibition zone assay on solid media was determined to evaluate the antimicrobial potential against the bacteria. *Escherichia coli* and *Staphylococcus aureus* are purified and stored at low temperatures. Firstly, after the bacteria were activated, 100 µL of the diluted bacteria solution was evenly coated on the Mueller–Hinton agar plates. Next, the 13 mm diameter discs (S = 132.7 mm^2^) obtained by cutting the film with a circular knife were placed on the agar plate, and the bacteria were allowed to grow fully for 24 h at 37 °C. Finally, the antibacterial ability was evaluated by observing the bacteriostatic zone.

### 2.5. Statistical Analysis

Statistical analysis was performed by one-way analysis of variance (ANOVA), and all the quantitative data were expressed as mean ± standard deviations with *n* ≥ 3.

## 3. Results and Discussion

### 3.1. Morphology Analysis of LCNF 

**Figure 3:**
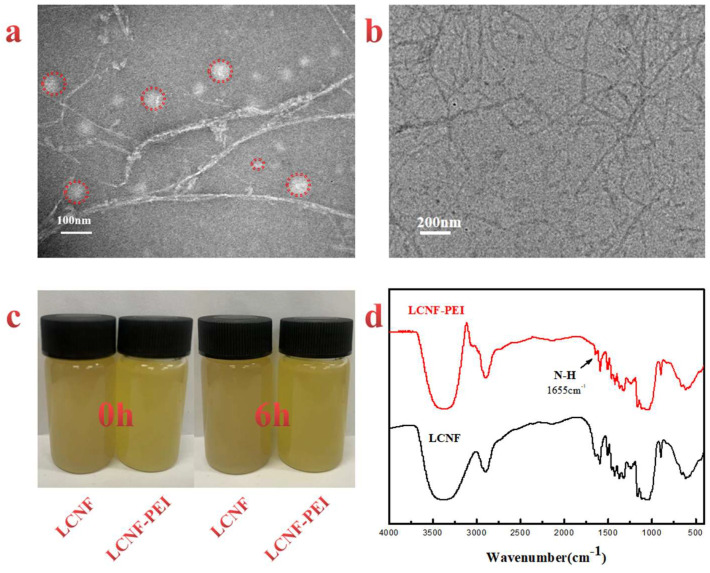
(**a**) The morphology of the LCNF suspension; (**b**) the morphology of the LCNF-PEI suspension; (**c**) digital camera images of LCNF and LCNF-PEI at different storage times; (**d**) the FTIR spectrum of the LCNF and LCNF-PEI.

Figure 3a illustrated the TEM images of a dilute suspension of LCNF. It can be observed that LCNF consisted of long nanofibrils with average diameters of 12–15 nm and sphercal nanolignins ranged from 5 to 40 nm. One part of lignin nanoparticles appeared evenly dispersion in the LCNF suspension, and other nanoparticles tend to coat the nanofibers to form complex structures. LCNF suspension was obtained by mechanical grinding after dilute alkali pretreatment, which has been proven to be an effective approach for the preparation of nanofibrils and nanolignins. The microstructure of LCNF–PEI can be observed from the Figure 3b. It can be found that the modified LCNF still maintained a nanometer scale, and there was no obvious agglomeration phenomenon. Figure 3c showed the stability of LCNF and LCNF–PEI at different storage times. The homogeneous suspension suggested that LCNF and LCNF–PEI had good dispersibility in aqueous system after 6 h, which did not sediment or flocculate. The modification of PEI did not reduce the dispersion performance of the LCNF suspension because of the electrostatic repulsion. Due to the chain-branching structure, PEI can provide a positive charge for nanoparticles, which is conducive to the stability of LCNF–PEI against agglomeration. The FTIR spectrum of the LCNF–PEI (Figure 3d) demonstrated distinct peaks from the amines, such as the N-H deformation at 1655 cm^−^^1^ [35]. This characteristic peak is ascribed to the success of PEI grafting.

### 3.2. Morphology Characterization of Nanocomposite Films

**Figure 4:**
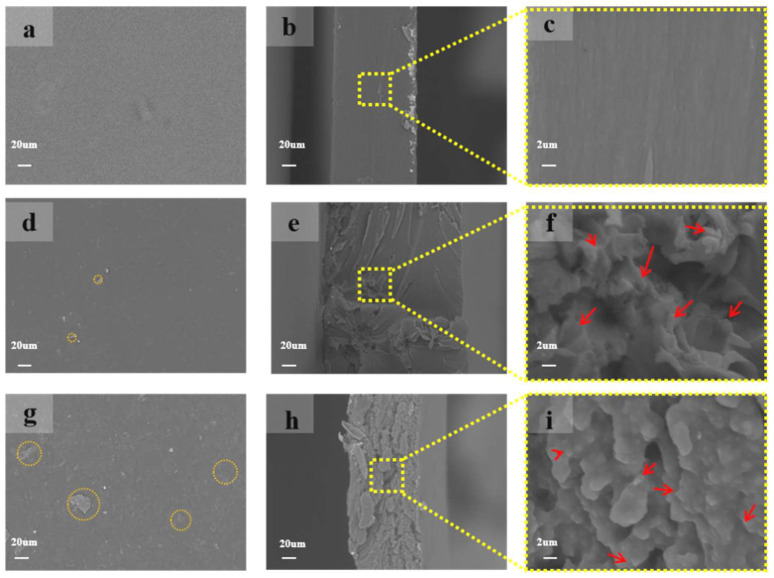
Morphology of (**a**) surface and (**b**,**c**) cross-section of PVA film, (**d**) surface and (**e**,**f**) cross-section of LPP1 film, (**g**) surface and (**h**,**i**) cross-section of LPP4 film.

SEM images of surface and fractured cross-section morphology of PVA, LPP1 and LPP4 are shown in Figure 4, and c, f and i represent the partial enlarged schematic diagrams of the dotted boxes in b, e and h, respectively. According to Figure 4a, it can be observed that the surface of PVA film is very smooth with no visual defects, indicating a homogeneous structure. When the amount of PEI-LCNF was 1 wt% (Figure 4d), LPP1 displayed a uniform and dense structure, indicating a good dispersion level of fillers for the nanocomposite films [36]. However, when the addition amount reached 4% (Figure 4g), LPP4 film became relatively rougher, corresponding to the aggregates of the LCNF. As highlighted by the circles in the figure, a large number of aggregation structures exist on the surface of the composite membrane. SEM images of the fractured cross sections of PVA, LPP1 and LPP4 obtained by stretching were shown in Figure 4b,e,h, and Figure 4c,f,i were local enlarged images of Figure 4b,e,h, respectively. As demonstrated in Figure 4b,c, the cross section of PVA still maintained a uniform and smooth structure and the thin film was directly broken under the action of axial tension, which indicated a brittle fracture behavior [37]. Compared with the PVA membrane, the cross section of LPP1 (Figure 4e) changed to become rougher. As can be observed from Figure 4f, LPP1 exhibited a porous and relatively uniform microstructure. It is difficult to observe the individual of filler dispersion in the PVA matrix due to its small particle size. The structure has obvious fibers pulling out phenomenon under the action of tensile stress to dissipate energy well, making the films have more excellent mechanical properties. When the addition amount was 4 wt% (Figure 4h), LPP4 exhibited irregular and coarse surface and occurred in macroscopic phase separation [38]. Moreover, some white points, corresponding to the large aggregates of the LCNF, could be observed at the fracture surface of LPP4.

### 3.3. Ultraviolet Resistance

**Figure 5:**
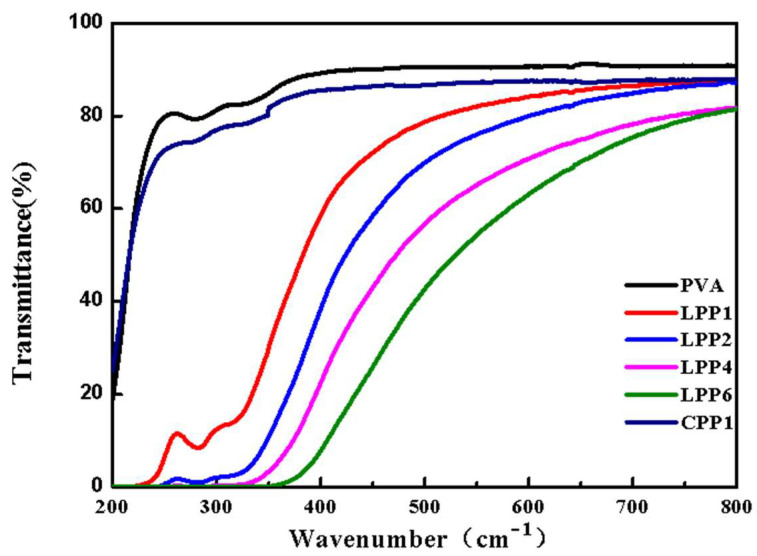
The UV-visible transmittance of the nanocomposite films.

The transmittance of the nanocomposite films in the wavelength range from 200 to 800 nm are shown in Figure 5. As can be observed, the pristine PVA has no shielding effect on the UV spectrum. After incorporated with LCNF–PEI, the films exhibited an attractive UV-blocking capacity and the UV-blocking efficiency increased with the increase in LCNF–PEI content. In the ultraviolet region of 200–400 nm, the transmittance of LPP1 in the 200–350 nm range was below 20%. There are obvious absorption peaks around 260 nm and 300 nm, which are typical characteristic peaks of benzene ring structure and the unconjugated phenolic hydroxyl [39]. When the amount of LCNF–PEI reached 6 wt%, an almost full UV shielding effect can be achieved. The remarkable UV-blocking performance of the material was mainly due to the π−π transition of the lignin guaiacyl structure [40]. With the increasing in the wavelength to the visible light region, the transmittance of all the films increased to above 50% at 500–800 nm, which indicated that the nanofillers were evenly dispersed in the PVA matrix. The addition of CNF without lignin did not significantly affect the transmittance of CPP1, and the curve was highly coincident with PVA. Therefore, it is deduced that the films have good barrier effect in the UV range due to the presence of the chromophores’ group of lignin. Moreover, the fillers filled up the free space in the polymer matrices, which eliminated the path for light transmission [41]. The addition of LCNF–PEI endows PVA films with excellent UV barrier performance.

### 3.4. Water Stability

**Figure 6:**
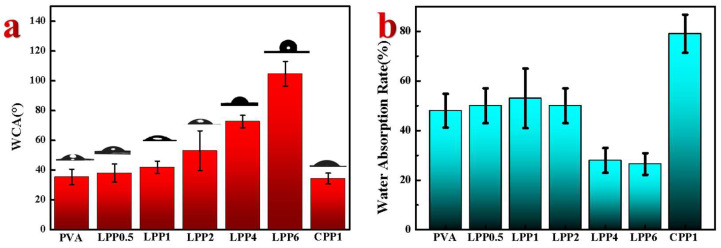
The water sensitivity of the films. (**a**) The water contact angle and digital photo of the film; (**b**) the water absorption rate of the films.

Figure 6a illustrates the effect of the LCNF–PEI content on the water vapor permeability for the films in distilled water. The WCA of the pure PVA films was 35°, which indicates that it has extremely hydrophilicity, mainly due to the abundant hydroxyl groups. Meanwhile, the introduction of hydrophilic groups into PVA is favorable for the enhancement of the surface hydrophilicity of the composite membrane, and the WCA of the CPP1 film with CNF was only 32°, just like the PVA film. Compared with the PVA film, the WCA of the LPP nanocomposite films was improved from 42° to 105°, with the effect of the incorporation of LCNF and the hydrophilic film gradually transformed into the hydrophobic film. The significant improvement is mainly ascribed to the benzene ring structure of residual lignin in LCNF, which has a certain hydrophobic ability. Meanwhile, the uniform dispersion of LCNF in the PVA matrix and increased roughness of hydrophobic surface may lead to the formation of a better micro/nano structure on the membrane surface, which reduces the surface energy and thus achieves the effect of hydrophobicity [42]. 

Figure 6b shows the effect of the LCNF–PEI content on the water uptake at equilibrium for the nanocomposite films. Compared with the water absorption rate of PVA (48.5%), the water absorption of LPP1 and LPP2 increased slightly (53.1% and 50%). This is because the surface of LCNF not only contains hydrophobic lignin, but also contains hydrophilic cellulose, which forms a competitive relationship. When the content of nanofillers was relatively low, the nanocomposite films still remained obvious hydrophilic effects. The water absorption of LPP6 decreased to 26.5% with the further increase in the LCNF addition, indicating an improvement in the water resistivity. Polymer interaction, which eliminated the hydrophilic functional groups, reduced the water sensitivity of the polymers [43], while the water absorption of CPP1 increased to 79.7% because of the introduction of the hydrophilic CNF. The stability of the composite film to water is highly consistent with the ratio of hydrophobic and hydrophilic groups. Therefore, LCNF can be used as an excellent hydrotropic agent to improve the hydrophobicity performance of bio-based nanocomposites. 

## 4. Mechanical Properties 

**Figure 7:**
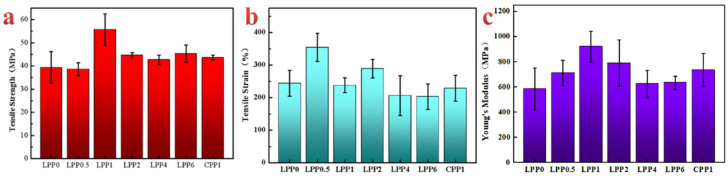
Mechanical properties of the films. (**a**) Tensile strength of the films; (**b**) tensile strain of the films; (**c**) Young’s modulus of the films.

The mechanical properties of the films are shown in Figure 7a–c. It can be observed that the LCNF content played an important role on the mechanical properties of the composites. The tensile strength (*σ_b_*) increased from 39.37 to 54.56 MPa with increasing nanofiller content from 0 to 1 wt%. This indicated that the uniform incorporation of LCNF into the PVA matrix resulted in strong interactions between fillers and matrix. This could be related to the stretching resistance of the polymer chain and nanofillers in the bonded points by hydrogen interaction. Meanwhile, mechanical interlocking also contributed to the phenomenon of “pulling out” in the process of tensile, thus the dissipating energy enhances the mechanical strength [21]. Then, the *σ_b_* values of nanocomposite films decreased with the further increasing filler content, but *σ_b_* of LPP6 still increased by about 20% compared with the pure PVA film. Meaningfully, compared with CPP1, the tensile strength of LPP1 (54.56 MPa) was larger than CPP1 (43.36 MPa), which indicated that the existence of lignin has a positive effect on the mechanical properties. Meanwhile, the elongation at break of LPP0.5 was significantly improved from 244.28% to 354.37%, which may be due to the intense interaction of LCNF–PEI and PVA [44], while by the further addition of LCNF–PEI, the increase in the elongation at break was inhibited, owing to the agglomeration phenomenon of nanofillers. As shown in the Figure 7c, the Young’s modulus of PVA was only 583.4 MPa. With the introduction of LCNF–PEI, the modulus of the films demonstrated a trend of increasing first and then decreasing. When the 1 wt% LCNF–PEI was added, the modulus of LPP1 increased by 58.0% to 922 MPa. Therefore, the unique rigid benzene ring structure in LCNF system is more conducive to the enhancement of tensile strength and stiffness, which restricted the motion of the matrix. 

### 4.1. Thermal Stability of the Nanocomposites

**Figure 8:**
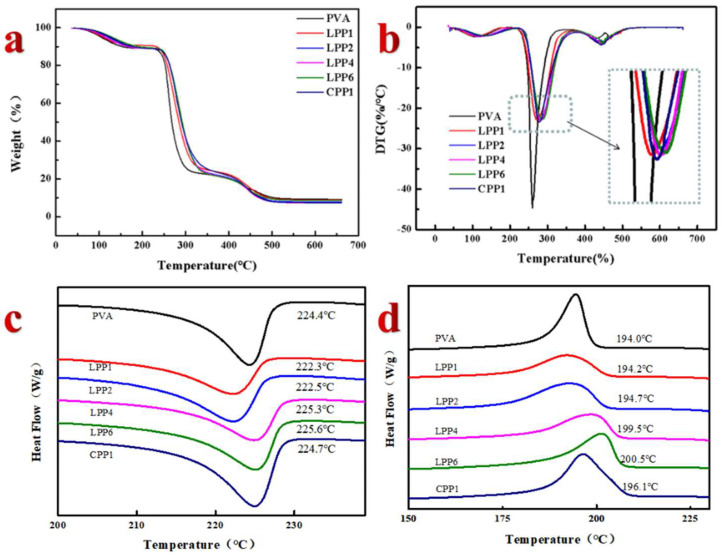
Thermal stability of films of PVA, LPP, CPP. (**a**) TG curves of the films; (**b**) DTG curves of the films; (**c**,**d**) DSC curves of thin films.

According to the TGA and DTG curves shown in Figure 8a,b, the thermal decomposition of each film can be divided into three stages. The first stage of minor weight loss at 50 °C to 130 °C was related to the evaporation of bound water in the molecular chain and degradation of oligomers [45]. At the same time, the second stage of rapid mass reduction occurred at 230–370 °C, and could be assigned to the degradation of the lignin and cellulose molecular chain side-chain. The weight loss peak at 400 °C to 500 °C for all films were believed to be from the decomposition of the main chain of PVA. It can be observed that the addition of LCNF–PEI had a positive effect on the thermal stability of composite films. The largest degradation temperatures (*T_max_*) of films increased from 260 °C to 283 °C with an increase in LCNF–PEI content from 0 to 6 wt%. This is mainly attributed to the formation of the thermal barrier of the stable aromatic units and intermolecular interactions between the PVA and nanofillers. Figure 8c,d shows the DSC curves of the composite films. We can clearly find that with the addition of LCNF–PEI, the melting temperature (*T_m_*) of the composite film first decreased and then increased. By comparing the *T_m_* of PVA (224.4 °C), LPP1 (222.3 °C) and CPP1 (224.7 °C), it can be concluded that the present of lignin hindered the hydrogen bonding between CNF and PVA, resulting in the decrease in *T_m_*. With the increase in LCNF–PEI, the interaction of the hydrogen bond between CNF and PVA bond becomes stronger, and *T_m_* can be continuously improved. The crystallization temperature (*T_c_*) increases with the increase in LCNF–PEI, which revealed rapid crystallization of PVA due to the introduction of LCNF as a nucleating agent [46].

### 4.2. Antibacterial Properties 

**Figure 9:**
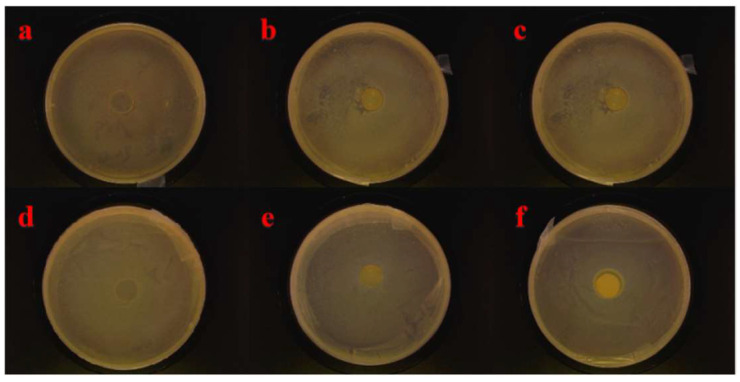
Antibacterial properties against *S. aureus* with (**a**) PVA, (**b**) LPP1, (**c**) LPP4 and *E. coli* with (**d**) PVA, (**e**) LPP1, (**f**) LPP4.

Antimicrobial properties of the composite films against both gram-positive (*S. aureus*) and gram-negative (*E. coli*) bacteria were performed through the inhibition zone test, and the obtained results were represented in Table 1 and Figure 9. We can observe that the PVA film was ineffective against both the bacteria. With the introduction of LCNF–PEI, there is a clear sterile zone around the membrane and both *S. aureus* and *E. coli* have similar and obvious inhibitory effects. The inhibition zone of the membrane gradually increased with the increase in LCNF–PEI content. The area increased from 146.0 ± 1.5 mm^2^ to 207.4 ± 2.3 mm^2^ with increasing nanofiller content from 1 to 4 wt% for *S. aureus*. Similarly, the area increased from 144.6 ± 3.1 mm^2^ (LPP1) to 212.2 ± 4.0 mm^2^ (LPP4) for *E. coli.* This is mainly because positive charges on the amine groups of PEI can be tightly bound to the bacterial membrane through ion exchange to achieve the effect of sterilization [33]. It can be concluded that the nanocomposite films were effective against both the bacteria due to the surface amine groups, and the antibacterial ability also increased with the enhancement of LCNF–PEI.

## 5. Conclusions

In summary, a series of PEI-modified LCNF-enhanced PVA nanocomposite films were prepared via the solvent casting method. Compared with the pure PVA film, the composite films have excellent thermal stability, tensile strength, hydrophobic performance, UV resistance and antibacterial performance. The tensile strength of LPP1 was 54.56 MPa, which was higher than those of the pure PVA film (39.37 MPa). The TGA results demonstrated that the thermal stability increased because of the formation of the thermal barrier of the stable aromatic units and intermolecular interactions between the PVA and LCNF. Lignin in LCNF endowed the films’ excellent UV shielding and moisture stability. Meanwhile, the PEI enhanced the antimicrobial properties against both *S. aureus* and *E. coli* bacteria because of the positive charges on the amine groups. Therefore, as value-added products, the LCNF–PEI/PVA nanocomposite films could have great applications in the field of the photosensitive and antimicrobial packaging industry. 

## Figures and Tables

**Table 1:** Antibacterial activity of different films.

Samples	The Area of the Inhibition Zone (mm^2^)	
	*Staphylococcus aureus*	*Esherichia coli*
PVA	132.7 ± 2.2 (a)	132.7 ± 1.3 (d)
LPP1	146.0 ± 1.5 (b)	144.6 ± 3.1 (e)
LPP4	207.4 ± 2.3 (c)	212.2 ± 4.0 (f)

## Data Availability

The data presented in this study are available upon request from the corresponding author.

## References

[1] Dou J., Vuorinen T., Koivula H., Forsman N., Sipponen M., Hietala S. (2021). Self-standing lignin-containing willow bark nanocellulose films for oxygen blocking and UV shielding. ACS Appl. Nano Mater..

[2] Abitbol T., Ahniyaz A., Álvarez-Asencio R., Fall A., Swerin A. (2020). Nanocellulose-based hybrid materials for UV blocking and mechanically robust barriers. ACS Appl. Bio Mater..

[3] Mamidi N., Velasco Delgadillo R.M. (2021). Design, fabrication and drug release potential of dual stimuli-responsive composite hydrogel nanoparticle interface. Colloids Surf. B.

[4] Mamidi N., Velasco Delgadillo R.M., Barrera E.V. (2021). Covalently Functionalized Carbon Nano-Onions Integrated Gelatin Methacryloyl Nanocomposite Hydrogel Containing γ-Cyclodextrin as Drug Carrier for High-Performance pH-Triggered Drug Release. Pharmaceuticals.

[5] Gaidukovs S., Platnieks O., Gaidukova G., Starkova O., Barkane A., Beluns S., Thakur V.K. (2021). Understanding the impact of microcrystalline cellulose modification on durability and biodegradation of highly loaded biocomposites for woody like materials applications. J. Poly. Environ..

[6] Zhang Y., Remadevi R., Hinestroza J.P., Wang X., Naebe M. (2020). Transparent ultraviolet (UV)-shielding films made from waste hemp hurd and polyvinyl alcohol (PVA). Polymers.

[7] Ji X., Guo J., Guan F., Liu Y., Yang Q., Zhang X., Xu Y. (2021). Preparation of Electrospun Polyvinyl Alcohol/Nanocellulose Composite Film and Evaluation of Its Biomedical Performance. Gels.

[8] Chen X., Taguchi T. (2020). Enhanced skin adhesive property of hydrophobically modified poly(vinyl alcohol) films. ACS Omega.

[9] Wang L., Hu J., Liu Y., Shu J., Wu H., Wang Z., Pan X., Zhang N., Zhou L., Zhang J. (2020). Ionic liquids grafted cellulose nanocrystals for high-strength and toughness PVA nanocomposite. ACS Appl. Mater. Inter..

[10] Jiang Y., Song Y., Miao M., Cao S., Feng X., Fang J., Shi L. (2015). Transparent nanocellulose hybrid films functionalized with ZnO nanostructures for UV-blocking. J. Mater. Chem. C.

[11] Kaur M., Santhiya D. (2020). UV-shielding antimicrobial zein films blended with essential oils for active food packaging. J. Appl. Polym. Sci..

[12] Yang M., Zhang X., Guan S., Dou Y., Gao X. (2020). Preparation of lignin containing cellulose nanofibers and its application in PVA nanocomposite films. Int. J. Biol. Macromol..

[13] Nair S.S., Yan N. (2015). Effect of high residual lignin on the thermal stability of nanofibrils and its enhanced mechanical performance in aqueous environments. Cellulose.

[14] Rojo E., Peresin M.S., Sampson W.W., Hoeger I.C., Vartiainen J., Laine J., Rojas O.J. (2015). Comprehensive elucidation of the effect of residual lignin on the physical, barrier, mechanical and surface properties of nanocellulose films. Green Chem..

[15] Xing L., Hu C., Zhang W., Guan L., Gu J. (2020). Biodegradable cellulose I (II) nanofibrils/poly (vinyl alcohol) composite films with high mechanical properties, improved thermal stability and excellent transparency. Int. J. Biol. Macromol..

[16] Beluns S., Gaidukovs S., Platnieks O., Gaidukova G., Mierina I., Grase L., Starkova O., Brazdausks P., Thakur V.K. (2021). From wood and hemp biomass wastes to sustainable nanocellulose foams. Ind. Crops Prod..

[17] Haque A.N.M.A., Zhang Y., Naebe M. (2021). A review on lignocellulose/poly (vinyl alcohol) composites: Cleaner approaches for greener materials. Cellulose.

[18] Wang Z., Gnanasekar P., Nair S.S., Farnood R.R., Yi S., Yan N. (2020). Biobased epoxy synthesized from a vanillin derivative and its reinforcement using lignin-containing cellulose nanofibrils. ACS Sustain. Chem. Eng..

[19] Bian H., Wei L., Lin C., Ma Q., Dai H., Zhu J. (2018). Lignin-containing cellulose nanofibril-reinforced polyvinyl alcohol hydrogels. ACS Sustain. Chem. Eng..

[20] Fortunati E., Yang W., Luzi F., Kenny J., Torre L., Puglia D. (2016). Lignocellulosic nanostructures as reinforcement in extruded and solvent casted polymeric nanocomposites: An overview. Eur. Polym. J..

[21] Chen Y., Fan D., Han Y., Lyu S., Lu Y., Li G., Jiang F., Wang S. (2018). Effect of high residual lignin on the properties of cellulose nanofibrils/films. Cellulose.

[22] Zhong Y., Gu L., Wang S., Jin Y., Xiao H. (2019). Green and superhydrophobic coatings based on tailor-modified lignocellulose nanofibrils for self-cleaning surfaces. Ind. Eng. Chem. Res..

[23] Poletto M., Zattera A.J., Forte M.M., Santana R.M. (2012). Thermal decomposition of wood: Influence of wood components and cellulose crystallite size. Bioresour. Technol..

[24] Zhang N., Tao P., Lu Y., Nie S. (2019). Effect of lignin on the thermal stability of cellulose nanofibrils produced from bagasse pulp. Cellulose.

[25] Liu X., Li Y., Ewulonu C.M., Ralph J., Xu F., Zhang Q., Wu M., Huang Y. (2019). Mild alkaline pretreatment for isolation of native-like lignin and lignin-containing cellulose nanofibers (LCNF) from crop waste. ACS Sustain. Chem. Eng..

[26] Jahed E., Khaledabad M.A., Bari M.R., Almasi H. (2017). Effect of cellulose and lignocellulose nanofibers on the properties of Origanum vulgare ssp. gracile essential oil-loaded chitosan films. React. Funct. Polym..

[27] Huang D., Wu M., Wang C., Kuga S., Huang Y. (2020). Effect of partial dehydration on freeze-drying of aqueous nanocellulose suspension. ACS Sustain. Chem. Eng..

[28] Huang P., Zhao Y., Kuga S., Wu M., Huang Y. (2016). A versatile method for producing functionalized cellulose nanofibers and their application. Nanoscale.

[29] Rajinipriya M., Nagalakshmaiah M., Robert M., Elkoun S. (2018). Importance of agricultural and industrial waste in the field of nanocellulose and recent industrial developments of wood based nanocellulose: A review. ACS Sustain. Chem. Eng..

[30] Bian H., Chen L., Dong M., Wang L., Wang R., Zhou X., Wu C., Wang X., Ji X., Dai H. (2021). Natural lignocellulosic nanofibril film with excellent ultraviolet blocking performance and robust environment resistance. Int. J. Biol. Macromol..

[31] Wang X., Jia Y., Liu Z., Miao J. (2018). Influence of the lignin content on the properties of poly(lactic acid)/lignin-containing cellulose nanofibrils composite films. Polymers.

[32] Gibney K.A., Sovadinova I., Lopez A.I., Urban M., Ridgway Z., Caputo G.A., Kuroda K. (2012). Poly(ethylene imine)s as antimicrobial agents with selective activity. Macromol. Biosci..

[33] You M., Li W., Pan Y., Fei P., Wang H., Zhang W., Zhi L., Meng J. (2019). Preparation and characterization of antibacterial polyamine-based cyclophosphazene nanofiltration membranes. J. Membr. Sci..

[34] Maizura M., Fazilah A., Norziah M.H., Karim A.A. (2007). Antibacterial activity and mechanical properties of partially hydrolyzed sago starch-alginate edible film containing lemongrass oil. J. Food Sci..

[35] Mi H.-Y., Jing X., Zheng Q., Fang L., Huang H.-X., Turng L.-S., Gong S. (2018). High-performance flexible triboelectric nanogenerator based on porous aerogels and electrospun nanofibers for energy harvesting and sensitive self-powered sensing. Nano Energy.

[36] Katekhong W., Wongphan P., Klinmalai P., Harnkarnsujarit N. (2022). Thermoplastic starch blown films functionalized by plasticized nitrite blended with PBAT for superior oxygen barrier and active biodegradable meat packaging. Food Chem..

[37] Ogunsona E.O., Mekonnen T.H. (2020). Multilayer assemblies of cellulose nanocrystal-polyvinyl alcohol films featuring excellent physical integrity and multi-functional properties. J. Colloid Interf. Sci..

[38] Phothisarattana D., Wongphan P., Promhuad K., Promsorn J., Harnkarnsujarit N. (2021). Biodegradable Poly (Butylene Adipate-Co-Terephthalate) and Thermoplastic Starch-Blended TiO_2_ Nanocomposite Blown Films as Functional Active Packaging of Fresh Fruit. Polymers.

[39] Zhang Y., Haque A.N.M.A., Naebe M. (2021). Lignin-cellulose nanocrystals from hemp hurd as light-coloured ultraviolet (UV) functional filler for enhanced performance of polyvinyl alcohol nanocomposite films. Nanomaterials.

[40] Xiong F., Han Y., Wang S., Li G., Qin T., Chen Y., Chu F. (2017). Preparation and formation mechanism of renewable lignin hollow nanospheres with a single hole by self-assembly. ACS Sustain. Chem. Eng..

[41] Chatkitanan T., Harnkarnsujarit N. (2021). Effects of nitrite incorporated active films on quality of pork. Meat Sci..

[42] Klinmalai P., Srisa A., Laorenza Y., Katekhong W., Harnkarnsujarit N. (2021). Antifungal and plasticization effects of carvacrol in biodegradable poly (lactic acid) and poly (butylene adipate terephthalate) blend films for bakery packaging. LWT.

[43] Wongphan P., Khowthong M., Supatrawiporn T., Harnkarnsujarit N. (2022). Novel edible starch films incorporating papain for meat tenderization. Food Packag. Shelf Life.

[44] Harnkarnsujarit N., Li Y. (2017). Structure–property modification of microcrystalline cellulose film using agar and propylene glycol alginate. J. Appl. Polym. Sci..

[45] Zhang C., Nair S.S., Chen H., Yan N., Farnood R., Li F. (2020). Thermally stable, enhanced water barrier, high strength starch bio-composite reinforced with lignin containing cellulose nanofibrils. Carbohyd. Polym..

[46] Jang J.-H., Lee S.-H., Endo T., Kim N.-H. (2015). Dimension change in microfibrillated cellulose from different cellulose sources by wet disk milling and its effect on the properties of PVA nanocomposite. Wood Sci. Technol..

